# Comparative Genome and Network Centrality Analysis to Identify Drug Targets of *Mycobacterium tuberculosis H37Rv*

**DOI:** 10.1155/2015/212061

**Published:** 2015-11-05

**Authors:** Tilahun Melak, Sunita Gakkhar

**Affiliations:** ^1^Department of Computer Science, Dilla University, P.O. Box 419, Dilla, SNNPR, Ethiopia; ^2^Department of Mathematics, IIT Roorkee, Roorkee, Uttarakhand 247667, India

## Abstract

Potential drug targets of *Mycobacterium tuberculosis H37Rv* were identified through systematically integrated comparative genome and network centrality analysis. The comparative analysis of the complete genome of *Mycobacterium tuberculosis H37Rv* against Database of Essential Genes (DEG) yields a list of proteins which are essential for the growth and survival of the pathogen. Those proteins which are nonhomologous with human were selected. The resulting proteins were then prioritized by using the four network centrality measures: degree, closeness, betweenness, and eigenvector. Proteins whose centrality value is close to the centre of gravity of the interactome network were proposed as a final list of potential drug targets for the pathogen. The use of an integrated approach is believed to increase the success of the drug target identification process. For the purpose of validation, selective comparisons have been made among the proposed targets and previously identified drug targets by various other methods. About half of these proteins have been already reported as potential drug targets. We believe that the identified proteins will be an important input to experimental study which in the way could save considerable amount of time and cost of drug target discovery.

## 1. Introduction

*Mycobacterium tuberculosis* (*Mtb*), the etiological agent of tuberculosis (TB), is the second main cause of death and infection for human among infectious diseases next to Human Immunodeficiency Virus (HIV) [1] and* Mycobacterium tuberculosis H37Rv* is the most studied strain. According to WHO global tuberculosis report of 2013, there were an estimated 8.6 million new cases and 1.3 million TB deaths in 2012 [2]. The estimate also showed that 3.6% of the new and 20.2% of previously treated cases are multidrug-resistance* tuberculosis* (MDR-TB) cases. Even though the current frontline anti-*Mycobacterium* drugs are mainly responsible for controlling and treatment of the disease to the extent that is being existing today, they have several shortcomings [3]. The main of them is the emergence of MDR-TB and extensively drug-resistant tuberculosis (XDR-TB) which could be able to render even these frontline drugs inactive. Some of the drugs like rifampicin have adverse side effects which lead to patient compliance. Most of these drugs are not also effective in acting on the latent forms of* Bacillus*. The need for careful consideration of vicious interactions between TB and HIV during drug discovery process for* Mtb* extends the challenge further [4].

The stated challenges and limitations of the existing frontline antibiotics for* Mtb* led to exhaustive computational and experimental methods to identify potential new drug targets for the pathogen. The stream which focuses on identifying the essential genes for the survival and growth of the pathogen is one of them. There are three main findings which proposed the lists of essential genes for the survival and growth of* Mycobacterium tuberculosis H37Rv* [5–7]. These findings were compiled and stored in Database of Essential Genes (DEG) for the intended users [8–10]. The database has been used to propose potential drug targets of* Mycobacterium tuberculosis H37Rv* [11]. In the study, the complete genome of* Mycobacterium tuberculosis H37Rv* was blasted against DEG to identify essential genes and the resulting dataset was further analyzed for similarity search against human genome to identify genes which are not similar with human to avoid host toxicity. Since two of the main findings about the essential genes were published after this study, it is possible to hypothesise that a comprehensive set of potential drug targets of* Mycobacterium tuberculosis H37Rv* could be obtained through a systematic computational analysis on the integrated dataset from DEG which incorporates those recent findings.

Generally, computational methods identify a larger number of potential drug targets which could be difficult to experimentally validate all of the targets due to time and cost constraints. Our main objective in this study is to identify and prioritize the potential drug targets of* Mycobacterium tuberculosis H37Rv* by integrating the analysis of comparative genome and network centrality measures of protein-protein interaction network of the pathogen. The stated limitation with respect to the global network centrality measures is that they are mainly based on only shortest paths [12]. Even though nonshortest paths could be important while spreading information in the cellular network, the shortest paths yield a higher coverage than observed directly neighbours locally from protein interaction data. It has also been hypothesised that shortest paths are the most feasible paths that can be taken by proteins to communicate with each other [13].

In this paper, a list of 137 potential drug targets of* Mycobacterium tuberculosis H37Rv* has been identified. These proteins are essential for the growth and survival of the pathogen, nonhomologous with human and prioritized based on their network centrality measure values where all of them are found within the close neighbourhood of the centre of gravity of protein-protein interaction network. It has been found out that almost half of these proteins have been already reported as potential drug targets of the pathogen by other methods. The structural assessment showed that 28 out of the 137 (20.44%) proteins have solved structure.

## 2. Materials and Method

### 2.1. Comparative Analysis

The complete genome sequence dataset of* Mycobacterium tuberculosis H37Rv* was retrieved from Tuberculosis Database which is an integrated platform providing access to genome sequence, expression data, and literature curation for tuberculosis research [1, 14]. BLAST search of the retrieved protein coding genes was carried out against DEG to identify essential genes. The corresponding protein sequences obtained after DEG search were subjected to a BLASTp against the nonredundant database with an *e*-value threshold cut-off set to 0.005 [15]. The search was also restricted to* H. sapiens* because the objective was to find only those proteins, which do not have detectable human homologues to prevent host toxicity.

### 2.2. Network Analysis

#### 2.2.1. Statistical Network Properties

The protein-protein interaction network of* Mycobacterium tuberculosis H37Rv* was retrieved from Search Tool for the Retrieval of Interacting Genes/Proteins (STRING) database [16]. The interactome network could contain false positives and false negatives which might affect the quality of the dataset and have an impact on the result. Interactions labeled with only “medium confidence” and “high confidence” scores were considered to minimize this impact. The statistical properties of the generated proteome network were characterized by different measures such as degree distribution, characteristic path length, and clustering coefficient to understand the general functional organization of interacting proteins.

The degree or connectivity *k* of a given network is equal to the number of connected neighbours or adjacent nodes. The degree distribution *p*(*k*), which has become one of the most prominent characteristics of network topology, is the measure of the proportion of nodes in the network having degree *k* [17].

For any two nodes *n*_*i*_ and *n*_*j*_ in a network with *n* vertices, the distance *d*_*ij*_ between them is defined as the length of the* shortest path* between the vertices, that is, the minimal number of edges that need to be traversed to travel from vertex *n*_*i*_ to *n*_*j*_. The path between two nodes does not necessarily have to be unique since there could be several alternative paths with the same path length. The characteristic path length is defined as the average shortest path of overall pairs of nodes in the network with *n* vertices [17].

Another important property of the network which shows local cohesiveness is the* clustering coefficient C* [17]. It is a measure of the probability that two nodes with a common neighbour to be connected. It is an indicator of the internal structure of the network. In undirected network, for a given node *n*_*i*_ with *k*_*i*_ neighbours, there exist *E*_max_ = *k*_*i*_(*k*_*i*_ − 1)/2 possible edges between the neighbors. Clustering coefficient *c*_*i*_ of vertex *n*_*i*_ is then given as the ratio of the actual number of edges *E*_*i*_ between the neighbors to the maximal number *E*_max_:

(1) $$\begin{array}{c} c_{i}=\frac{2E}{k_{i}\left(k_{i}-1\right)}. \end{array}$$

The global or mean clustering coefficient *C*_*i*_ of the network is the average cluster coefficient of all vertices.

#### 2.2.2. Network Centrality

The resulting lists of proteins were further prioritized based on the four network centrality measures, namely, degree, closeness, betweenness, and eigenvector. The goal of these network centrality measures is to numerically characterize the importance of proteins in the biological system since centrality indices are used to quantify the nodes or edges that are more central than others.

For undirected Graph G having adjacency matrix *A* = (*A*_*ij*_), the degree centrality *k*_*i*_ of its *i*th node is given by

(2) $$\begin{array}{c} k_{i}=\overset{n}{\underset{j=1}{\sum }}A_{ij}. \end{array}$$

Closeness centrality of a node *s* is calculated as the inverse of the sum of distances from all other *t* nodes:

(3) $$\begin{array}{c} c_{clo}\left(\;s\;\right)=\frac{1}{\sum dist\left(s,t\right)}. \end{array}$$

The betweenness centrality is a measure of the total number of shortest paths between two nodes passing through the specified node.

Let *σ*_*st*_ be the number of shortest paths from *s* to *t* and *σ*_*st*_(*p*) denotes the number of shortest paths from node *s* to node *t* passing through *p*; then betweenness centrality *B*(*p*) of the node *p* is given by

(4) $$\begin{array}{c} B\left(\;p\;\right)=\underset{s\neq p\neq t}{\sum }\frac{\sigma_{st}\left(p\right)}{\sigma_{st}}. \end{array}$$

Let *X* = (*x*_1_, *x*_2_,…, *x*_*n*_) be an eigenvector of the adjacency matrix *A* with eigenvalue *λ*:

(5) $$\begin{array}{c} \lambda X=AX. \end{array}$$

The eigenvector centrality is given by

(6) $$\begin{array}{c} X_{i}=\lambda^{-1}\overset{n}{\underset{j=1}{\sum }}A_{ij}x_{j}. \end{array}$$

By Perron-Frobenius theorem, there is only one eigenvector *x* with all centrality values nonnegative and this is the unique eigenvector that corresponds to the largest eigenvalue *λ* [18].

**Figure 1:**
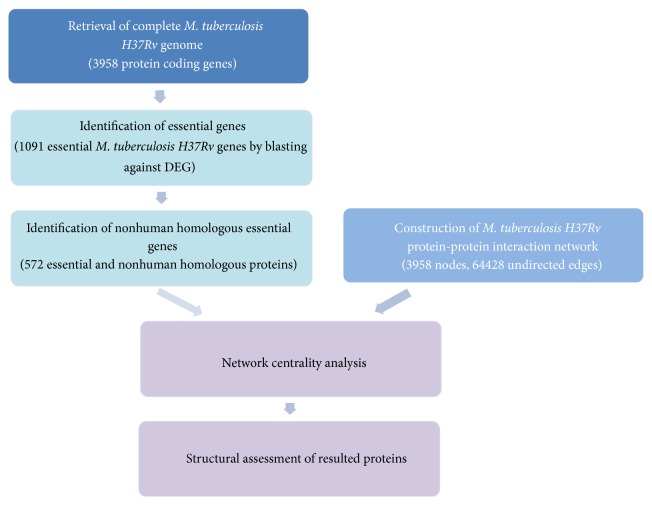
Progression of experiments. Different aspects indicated in this diagram are identification of essential genes, comparative analysis, construction of protein-protein interaction network, and network centrality analysis and validation.

Degree centrality is the most simple but also the most basic centrality measure which is used to identify an important node involved in a large number of interactions. It is a local centrality measure since determined by the number of its neighbors. It has been widely used for the analysis of biological networks [17]. Proteins with high degree centrality values are more likely to be essential for the survival and growth of the organism than proteins with low degree centrality values. In closeness centrality the specified nodes closeness to all other nodes of the network is quantified. An important node is typically close which means it can communicate quickly with the other nodes of the network. The betweenness centrality measure is a means to quantify the influence of a node in the interaction network. It shows that an important node lies on a high proportion of paths between other nodes in the interaction network. The eigenvector centrality of a node is directly dependent on the centrality values of its connected neighbors which means eigenvector centrality of each node is assigned a centrality value based on not only the quantity of its connections, but also their qualities. A high centrality value of the neighbors should result in a high centrality for the node under consideration. So the main idea in eigenvector centrality measure is that an important node is the one which is connected to important neighbors. The progression of the experiments in this study has been shown in Figure 1.

## 3. Results and Discussion

### 3.1. Comparative Analysis for Identifying Nonhomologous Essential Genes

The retrieved complete genome sequence dataset of* Mycobacterium tuberculosis H37Rv* consists of sequences of 3958 protein coding genes. These genes were then blasted against DEG to obtain essential genes. These genes are those which are indispensable for the survival and growth of the pathogen. As a result their functions are, therefore, considered a foundation of life. Defining these protein coding genes which are essential for the bacterial growth and its survival is believed to be important in identifying both key biological processes and potential targets for rational drug development [7]. A total of 1091 genes were identified as essential genes from the analysis.

One of the important questions that needs to be addressed while choosing potential drug targets for pathogens like* Mycobacterium tuberculosis H37Rv* is validating whether the potential proteins to be targeted are all absent in the host* H. sapiens* and therefore unique to the pathogen. Identifying those enzymes from the pathogen which does not share a similarity with the host proteins ensures that the targets have nothing in common with the host proteins, thereby eliminating undesired host protein-drug interactions. We have performed a comparative analysis of the host* Homo sapiens* and the pathogen* Mycobacterium tuberculosis* for the identified 1091 essential genes. We have adopted a stringent measure of listing out only those enzymes which have no similarity or negligible similarity (above the *e*-value threshold of 0.005) to the host proteins. With the aid of this approach 572 out of 1091 proteins are absent in the host* H. sapiens* and therefore they are unique to* Mycobacterium tuberculosis H37Rv*.

### 3.2. Interactome Analysis for Prioritizing Nonhomologous Essential Genes

#### 3.2.1. General View of* Mycobacterium tuberculosis H37Rv* Proteome Network

A proteome-scale interaction network of proteins in* Mycobacterium tuberculosis H37Rv* was generated from STRING database which is claimed to be a database and web resource dedicated to protein-protein interactions, including both physical and functional interactions [16]. The interactions are weighted and integrated from various sources like experimental repositories, computational prediction methods, and public text collections which makes it an acting comprehensive metadatabase that maps all interaction evidence onto a common set of genomes and proteins. In this database, a combined score has been assigned for each protein-protein linkage based on the evidence from various sources. A higher score is assigned for interactions which are supported by several types of evidence. Generally, these scores are broadly classified into three, namely, “low-scores” for value less than 0.4, “medium-scores” for values between 0.4 and 0.7, and “high-scores” for those associations whose values are greater than 0.7. The existence of false positives and false negatives is widely anticipated in the networks of these types which are being constructed by using the currently available methods [13]. A recent comprehensive study has also indicated that the protein-protein interaction networks generated from STRING database are of low quality consisting of a significant amount of false positives and false negatives [19]. All interactions with value of “low-scores” have been removed from this study to minimize the impact of the problem. The resulting network contains 64,428 interactions among 3,958 proteins. Of the total 64,428 interactions, 22,395 were labeled as “high-score” and 42,033 as “medium-score.” Despite of its shortcomings, this network provides a good framework for navigation through the proteome and it also allows for refinement of the network upon the availability of new experimental data.

**Figure 2:**
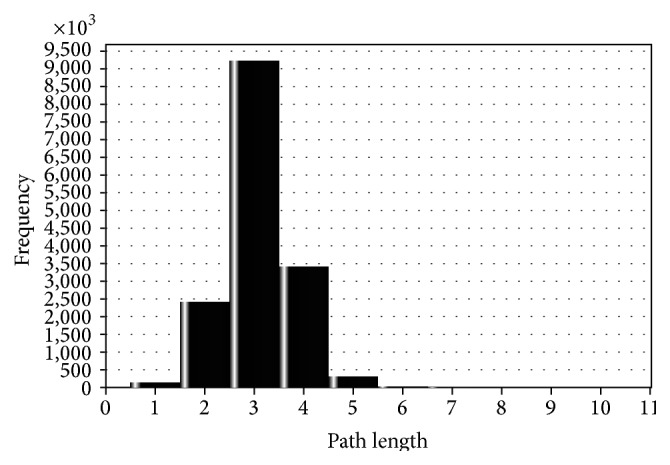
Shortest path length distribution. Distribution of shortest path lengths between reachable pairwise protein interactions.

**Figure 3:**
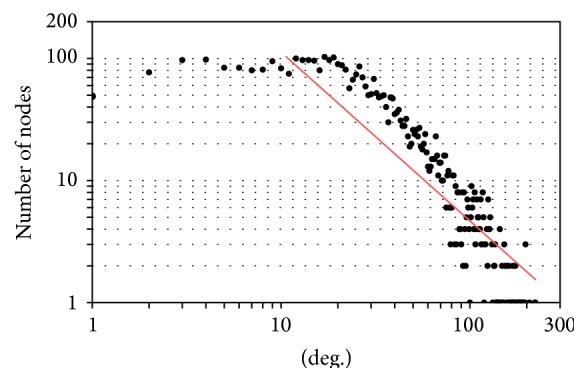
Node degree distribution. The distribution of the probability *p*(*k*) that the degree of a randomly chosen vertex equals *k* has been shown and it follows a power law *p*(*k*) ~ *k*.

Statistical properties of the generated network have been shown in Table 1 to describe its essential properties. The characteristic path length of the network, which is the average distance between all pairs of nodes, is smaller than log⁡(*n*). This implies that the* Mycobacterium tuberculosis H37Rv* proteome interaction network has “small world property” [20]. This property provides an idea about the network's navigability by indicating how fast information can be communicated in the system irrespective of the number of nodes. Thus, from this small world property of the network, we can understand that the network is efficient in the communication of biological information. This means one protein can have an influence on another with only a small number of intermediate reactions. The shortest path length distribution between pairwise protein interactions has been shown in Figure 2. As the degree distribution of the resulting network has also been shown in Figure 3, it exhibits scale-free property like many biological networks in which the degree distribution of proteins approximates a power law *p*(*k*) = *k* ^−*γ*^, with the degree exponent *γ* ~ 1.38. So there are very rare highly connected nodes called hubs in a vast majority of nodes with only a few connections. The* clustering coefficient* of the resulting network is significantly higher than the* clustering coefficient* of a random graph with the same number of vertices (0.008).

#### 3.2.2. Network Centrality Analysis

**Figure 4:**
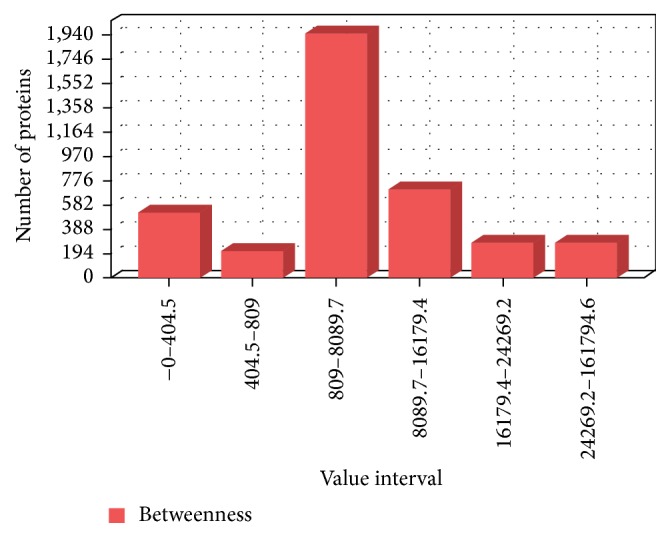
Distribution of betweenness centrality values. Longitudinal comparison of centrality values which shows the number of proteins located in separate score intervals based on betweenness centrality measure.

**Figure 5:**
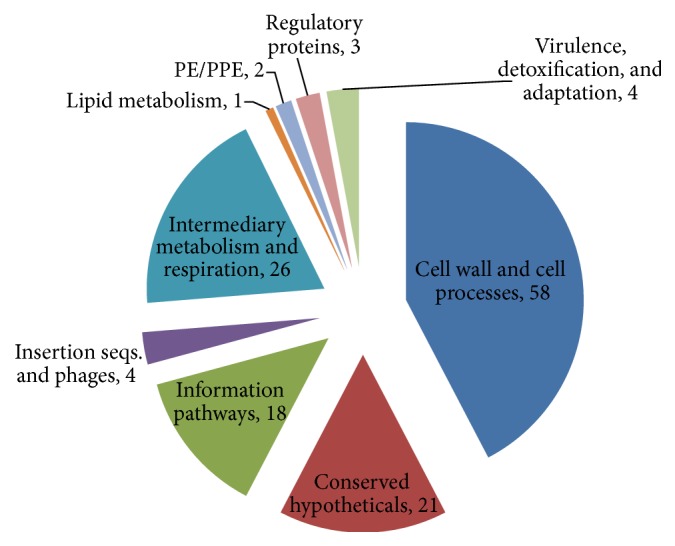
It illustrates the comprehensive list of central, nonhomologous, and essential proteins as potential drug targets. The candidate lists have been classified into their high level functional class and the distribution has been indicated in the diagram.

Comparative genome analysis was helpful in filtering out 572 nonhomologous and essential proteins of* Mycobacterium tuberculosis H37Rv*. However, this set is still very large to validate with the aid of experimental methods. The network centrality measures have been used for further ranking and prioritizing these proteins in the generated proteome interactome network. The objective was to order the proteins such that the most important proteins can be used first in an experiment. This has been done with the subsequent steps of sorting all of the proteins in the generated network, filtering proteins that are found near to the center of gravity and identifying the ordered list from nonhomologous and essential proteins which are found in the filtered list. For longitudinal comparison of centralities, the distribution of betweenness value of sorted proteins has been indicated in Figure 4. The diagram shows the number of proteins located in separate score intervals of the network. Betweenness centrality metric is one of the significant indicators of network essentiality because proteins with high betweenness are essential for the functioning of the system by serving as a bridge of communication between several other proteins in the network [21]. In this investigation, we tried to identify proteins which are found near to the centre of gravity of the proteome network by being connected with influential proteins. Since the characteristic path length of the generated network is 3.096, a protein is said to be at the centre of gravity if its betweenness measure is above the total number of shortest paths expected to pass through the protein in the functional network of interest, which is 12253.968. This criterion has been effectively used by Mazandu and Mulder in identification of potential drug targets of* Mycobacterium tuberculosis* [22]. With the aid of this principle we have got 137 ranked, essential, nonhomologous, and central proteins which we believe to be reliable targets for* Mycobacterium tuberculosis H37Rv*. The detailed list of these potential drug targets incorporating the network centrality measure scores and validation is provided as Supplementary Material available online at http://dx.doi.org/10.1155/2015/212061. The lists of refined targets were further categorized by high level of functional classes and the distribution of these potential drug targets per functional class is shown in Figure 5. The distribution indicated that most of the candidate drug targets are involved in cell wall and cell processes, followed by a significant proportion of proteins in intermediary metabolism and respiration, conserved hypothetical, and those belong to information pathway.

**Figure 6:**
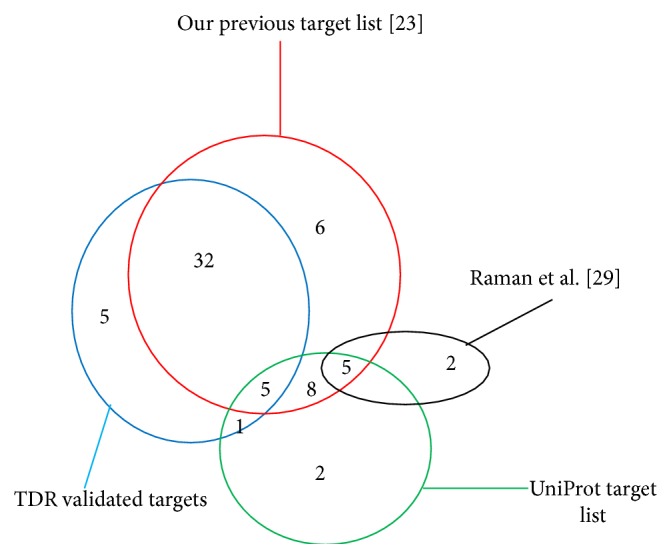
Venn diagram of proteins in the proposed list that are reported by other methods.

The resulting lists of candidate proteins were assessed by comparing with some of known drug targets as well as potential targets predicted by using different computational and experimental methods. The dataset for this purpose was obtained by integrating manually curated targets from TDR, high confidence targets from UniProt, and attractive targets obtained by Raman et al. [29] through a series of comprehensive filters. We have also used the potential drug targets list identified in our previous investigation [23]. The Venn diagram (Figure 6) shows the overlaps among these lists of drug targets and the proposed potential target list. Based on this assessment, 43 proteins in the list were TDR validated targets, 6 of which were in the UniProt target list. An additional of 18 proteins in our list were overlapped with UniProt's list, 5 of which were also predicted by Raman et al.; their list contains 2 more proteins. From our previous report 56 proteins were overlapped with the current candidates; some of them were already reported as potential targets by other methods. Moreover, there are four known targets of existing antitubercular drugs within this set. They are Rv1908c (KatG) (ranked 12th in the proposed list), Rv3795 (EmbB) (ranked 15th), Rv3793 (ranked 25th), and Rv3794 (ranked 30th). Rv1908c (KatG) is a validated drug target of Isoniazid whereas Rv3793, Rv3794 are target proteins of Ethambutol [24]. Rv3795 (EmbB) is a drug target for Rifampin, Isoniazid, and Ethambutol. Therefore, 67 (48.9%) proteins from our proposed list have been previously predicted or reported to be drug targets by the stated methods.

The lists of top 20 proteins according to each of the four centrality measures have been obtained. From these lists, 10 of the proteins are found to be common and they are listed in Table 2. It is hypothesised that these proteins are better targets since they have been identified in higher ranks of the four centrality measures of the interactome network. Additional information about each protein such as function, gene name, whether it has been reported as a drug target by other methods, and interaction with the host can be referred from the Supplementary Material.

Additionally, potential drug targets of the pathogen that interact with the host have been identified to understand the infection mechanism using a dataset obtained from a computational prediction of* Homo sapiens-Mycobacterium tuberculosis H37Rv* protein-protein interactions [25]. This dataset is thought as a golden dataset for host-pathogen interaction. As it has been shown in Table 3, 15 proteins from the proposed target lists interact with human. The reason for the presence of only few overlaps could be due to the fact that the host-pathogen interaction dataset is not comprehensive or the host interacting proteins are not necessarily essential to the pathogen and nonhomologous with human. Identifying proteins of the pathogen participating in the complex interplay with the host could significantly increase the reliability of the targets since these interactions are key factors in determining the outcome of the infection [26].

Further,* the study of Mycobacterium tuberculosis* virulence is another path which has got much attention in the design of drugs with a new mechanism of action, the production of modern concepts, and tuberculosis treatment schemes [27]. Virulence factors have evolved as a response to the host immune reaction. In recent times, many* mycobacterial* virulence genes that are essential for the virulence of* Mycobacterium tuberculosis complex* (*MTBC*) species have been reported by a number of studies. Most of these genes either encode enzymes of several lipid pathways, cell surface proteins, regulators, and proteins of signal transduction systems or are involved in* mycobacterial* survival inside the aggressive microenvironment of the host macrophages. We took a compiled list of virulence genes from Forrellad et al. [27] and tried to observe the overlap with our proposed potential targets. It has been found out that five genes from the proposed potential target list are also reported as virulence genes. These genes have been shown in Table 4.

### 3.3. Structural Assessment

One of the main criteria which increase the targetability of the prioritized lists of proteins is the availability of crystal structures. The Protein Data Bank (PDB) is freely accessible and the main worldwide repository for the three-dimensional structural data of biological macromolecules such as proteins and nucleic acids which is typically obtained by X-ray crystallography or NMR spectroscopy and submitted by biologists and biochemists from around the world [28]. By excluding those proteins which have more than 70% sequence identity, only 229 (about 6%) of* Mycobacterium tuberculosis* proteins have solved structure in PDB [29]. Hence, we checked the availability of solved structures of the identified potential lists of targets and out of 137 proteins from our proposed target list, 28 were successfully mapped to 82 structures from PDB which is approximated to 20.44%. This list has also been shown in Table 5 including the corresponding centrality measure values and PDB IDs of structures. However, reliable structures of the pathogen could still be obtained by using theoretically calculated homology models.

## 4. Assessment of the Method

**Figure 7:**
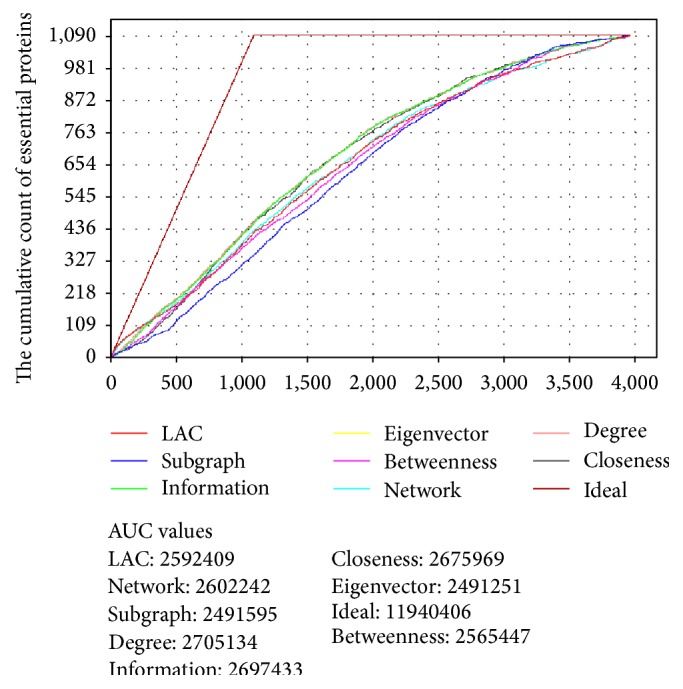
Jack-knife line chart of eight centrality measures. The cumulative count of essential proteins of eight different centrality measures has been shown to assess the performance of the four centrality measures used for this analysis.

It would be ideal to have standard validation data in order to assess the performance of the four centrality measures used in this analysis, but it is not readily available. The list of essential proteins obtained through a comparative analysis has been used as a test data. Since the main objective of centrality measures in a network is to identify the proteins which are influential, taking this data for evaluation is reasonable. These centrality measures were compared with other typical centrality measures: Local Average Connectivity- (LAC-) based method, Network Centrality (NC), Subgraph Centrality (SC), and Information Centrality (IC). As it can be seen in the jack-knife line chart (Figure 7), there is no huge difference among the eight centrality measures with the AUC value of degree centrality the highest of all. Information and closeness centralities ranked second and third, respectively.

Other testing data, including validated drug targets and intersection of high confidence targets from UniProt and attractive targets from Raman et al. [29], were identified. This list contains 47 proteins. Then, the eight centrality measures were compared in terms of the average rank of the drug targets in which lower average rank indicates better performance. The absolute count of drug targets in 1% of all candidate proteins (practically in the top 40 proteins), in the top 5% (practically in the top 198 proteins), in the top 10% (practically in the top 396 proteins), in the top 15% (practically in the top 594 proteins), and in the top 20% (practically in the top 792 proteins) among all candidates was reported (Table 6). For instance, in the top 1%, betweenness centrality identified 2 drug targets while the others found 1. Eigenvector identified joint maximum potential targets in all of top 5%, 10%, 15%, and 20%. We took up to 20% for comparison because these proteins are found near the center of gravity values.

## 5. Conclusion

In this study we have identified a list of proteins which could be an attractive and reliable target for* Mycobacterium tuberculosis H37Rv* through a comprehensive analysis of comparative genome and network centrality measures of protein-protein interaction network. The comparative genome analysis has helped in identifying those lists of proteins which were stated as essential for the survival and growth of the pathogen to increase success rate of drugs to be designed. It was also useful in filtering out those proteins which are present in human to eliminate all those with a risk of causing host toxicity. In traditional drug discovery the side effect or drug safety has normally been addressed by making modification on the drug molecule, but systematic way of dealing with this problem at the drug target identification phase in the modern rational drug discovery process seems to be more effective [13]. These refined lists of proteins were then analyzed by network centrality measures to prioritize the identified lists of candidate protein targets by hypothesising that the proteins that are at the centre of gravity of the disease specific protein-protein interaction network are more important proteins in the pathogen and hence more likely to be attractive targets. The comparison of these lists of targets with some of known drug targets as well as potential targets predicted by using different computational and experimental methods revealed that about half of them have been previously predicted or reported to be potential drug targets for* Mycobacterium tuberculosis H37Rv*. The structural assessment of these proteins has also showed those which have an experimentally solved three-dimensional structure which increases their targetability. In general, we believe that this comprehensive analysis will have significant contribution in providing an important input for the experimental study of developing new antibiotics for infamous* Mycobacterium tuberculosis H37Rv* pathogen.

## Supplementary Material

The list contains prioritized and detailed list of proteins proposed as potential drug targets for *Mycobacterium tuberculosis H37Rv*. The proteins are essential to the growth and survival of the pathogen, nonhomologous with the host and found in the centre of gravity of protein-protein interactome network. The corresponding function, structural information and validation if these proteins have been reported by other methods are also incorporated.

## Figures and Tables

**Table 1:** Network statistics.

Parameter	Value
Number of nodes (*n*)	3958
Connected components	8
Network diameter	10
Average number of neighbours	32.556
Network density	0.008
Network heterogeneity	0.942
Shortest paths	15519694 (99%)
Characteristic path length	3.096
Clustering coefficient	0.294

**Table 2:** Proteins in the top 20 of all of the four centrality measures.

Protein	Functional class	Network centrality scores				PDB
		Betweenness	Eigenvector	Degree	Closeness	
Rv1303	Cell wall and cell processes	115190.99	0.1066408	207	0.0490584	
Rv3019c	Cell wall and cell processes	71224.33	0.08889492	167	0.0488181	3H6P
Rv0311	Conserved hypotheticals	67942.516	0.07391167	156	0.048861504	
Rv0556	Cell wall and cell processes	66400.05	0.103606604	193	0.049006738	
Rv0451c	Cell wall and cell processes	66230.336	0.09615834	171	0.04883437	2LW3
Rv0288	Cell wall and cell processes	63475.453	0.09102694	170	0.048855472	2KG7
Rv0875c	Cell wall and cell processes	49197.566	0.11939961	197	0.04895823	
Rv1274	Cell wall and cell processes	46747.54	0.11324064	197	0.04900249	
Rv0817c	Cell wall and cell processes	40821.99	0.11914455	196	0.048973985	
Rv0358	Conserved hypotheticals	40230.57	0.07967692	154	0.048827738	

**Table 3:** Proposed targets which interact with the host.

Protein	Functional class	Betweenness	Eigenvector	Degree	Closeness
Rv1599	Intermediary metabolism and respiration	58334.63	0.002786182	82	0.048330363
Rv1908c	Virulence, detoxification, and adaptation	50548.035	0.005637622	76	0.048456423
Rv2150c	Cell wall and cell processes	37525.4	0.007632928	103	0.048433885
Rv3921c	Cell wall and cell processes	29409.086	0.006799817	68	0.048375268
Rv0732	Cell wall and cell processes	26715.15	0.011198077	111	0.04828495
Rv1415	Intermediary metabolism and respiration	23617.264	0.002809845	57	0.048159193
Rv2534c	Information pathways	22585.9	0.010742613	123	0.04837822
Rv2553c	Cell wall and cell processes	21154.973	0.004895513	53	0.04829615
Rv1602	Intermediary metabolism and respiration	20447.479	0.002363527	49	0.048105914
Rv1611	Intermediary metabolism and respiration	17798.758	0.002891334	57	0.048196144
Rv2455c	Intermediary metabolism and respiration	16318.1045	0.00160155	54	0.04806034
Rv3601c	Intermediary metabolism and respiration	15341.172	0.00251573	49	0.048123464
Rv2538c	Intermediary metabolism and respiration	14206.7705	0.002324578	65	0.0481065
Rv2987c	Intermediary metabolism and respiration	13133.913	0.001755499	52	0.04798341
Rv1712	Intermediary metabolism and respiration	12815.694	0.002191681	62	0.048019513

**Table 4:** Genes reported as virulence factors.

Gene name	Rv number	Functional class	Betweenness	Eigenvector	Degree	Closeness
sigA	Rv2703	Information pathways	56665.156	0.004549677	80	0.04838473
katG	Rv1908c	Virulence, detoxification, and adaptation	50548.035	0.005637622	76	0.048456423
icl1	Rv0467	Intermediary metabolism and respiration	23928.854	0.002939687	53	0.048157435
pafA	Rv2097c	Intermediary metabolism and respiration	19334.732	0.00727773	57	0.048266105
ideR	Rv2711	Regulatory proteins	15661.199	0.005902891	41	0.04817209

**Table 5:** Sorted lists of proteins proposed as potential drug targets which have solved structure.

Protein	Network centrality scores				PDB
	Betweenness	Eigenvector	Degree	Closeness	
Rv3019c	71224.33	0.08889492	167	0.0488181	3H6P
Rv0451c	66230.336	0.09615834	171	0.0488344	2LW3
Rv0288	63475.453	0.09102694	170	0.0488555	2KG7
Rv0058	57680.18	0.00275039	62	0.0481486	2R5U
Rv1908c	50548.035	0.00563762	76	0.0484564	1SFZ; 1SJ2; 2CCA; 2CCD; 4C50; 4C51
Rv2050	40088.305	0.01941196	91	0.0485105	2M4V; 2M6P
Rv2150c	37525.4	0.00763293	103	0.0484339	1RLU; 1RQ2; 1RQ7; 2Q1X; 2Q1Y; 4KWE
Rv3793	36733.14	0.06872068	136	0.0487904	3PTY
Rv2111c	35411.24	0.00444417	37	0.0479427	3M91; 3M9D
Rv0002	33375.504	0.00362037	90	0.0482796	3P16; 3RB9
Rv3597c	27758.719	0.01894281	73	0.048406	2KNG; 4E1P; 4E1R
Rv1837c	24302.383	0.00201247	47	0.0479375	2GQ3; 3S9I; 3S9Z; 3SAD; 3SAZ; 3SB0
Rv0467	23928.854	0.00293969	53	0.0481574	1F61; 1F8I; 1F8M
Rv3240c	22790.56	0.00369075	64	0.0481586	1NKT; 1NL3
Rv0216	20616.273	0.01939864	57	0.0482126	2BI0
Rv1611	17798.758	0.00289133	57	0.0481961	3QJA; 3T40; 3T44; 3T55; 3T78; 4FB7
Rv2773c	17351.877	0.00189925	52	0.0480259	1C3V; 1P9L; 1YL5; 1YL6; 1YL7
Rv0902c	16640.244	0.02111599	42	0.0480668	1YS3; 1YSR
Rv2711	15661.199	0.00590289	41	0.0481721	1B1B; 1FX7; 1U8R; 2ISY; 2ISZ; 2IT0
Rv3601c	15341.172	0.00251573	49	0.0481235	2C45
Rv2518c	14995.93	0.00281853	25	0.0477322	3VYN; 3VYO; 3VYP; 4GSQ; 4GSR; 4GSU; 4HU2; 4HUC
Rv0736	14264.871	0.01536277	50	0.0483009	3HUG
Rv2538c	14206.7705	0.00232458	65	0.0481065	3QBD; 3QBE
Rv3808c	13873.782	0.00814938	51	0.0480131	4FIX; 4FIY
Rv2416c	13419.646	0.00771849	39	0.0482891	3R1K; 3RYO; 3SXO; 3UY5
Rv2987c	13133.913	0.0017555	52	0.0479834	3H5E; 3H5H; 3H5J
Rv2986c	13109.861	0.00315177	40	0.0481223	4DKY; 4PT4
Rv2391	12401.702	0.00120635	45	0.0478749	1ZJ8; 1ZJ9

**Table 6:** Number of drug targets and its average position among different methods in top 1%, 5%, 10%, 15%, and 20% of the candidate proteins list.

Method	1% (40)	5% (198)	10% (396)	15% (594)	20% (792)
LAC	0	6	9	12	15
Subgraph	1	8	13	19	23
Information	1	6	9	13	17
Network	1	6	9	12	15
Eigenvector	1	8	13	19	23
Betweenness	2	7	10	14	18
Degree	1	6	9	13	17
Closeness	2	5	10	15	22
